# Impact of US funding cuts and stop work orders on TB services and research in South Africa

**DOI:** 10.5588/ijtldopen.25.0168

**Published:** 2025-04-09

**Authors:** N. Ndjeka, M. Kubjane, F. Abdullah, E. Mohr-Holland, P. Subrayen, M. Loveday, M. Dube, J. Boffa

**Affiliations:** ^1^ TB Cluster, South African National Department of Health, Pretoria, South Africa;; ^2^ South African TB Think Tank, Johannesburg, South Africa;; ^3^ Health Economics and Epidemiology Research Office (HE2RO), University of Witwatersrand, Johannesburg, South Africa;; ^4^ Office of AIDS and TB Research, South African Medical Research Council, Cape Town, South Africa;; ^5^ Departments of Public Health Medicine & Infectious Diseases, University of Pretoria, Pretoria, South Africa;; ^6^ Community Services and Health, City of Cape Town, Cape Town, South Africa;; ^7^ The Aurum Institute, Johannesburg, South Africa;; ^8^ HIV and other Infectious Diseases Research Unit (HIDRU), South African Medical Research Council, Durban, South Africa.

**Keywords:** tuberculosis, National TB Programme, USAID, CDC, MDR-TB

Dear Editor,

Over the past decade, South Africa has made great strides and reduced the incidence of TB by 57%.^1^ This inspiring reduction was driven by improved TB testing and treatment and the scale-up of antiretroviral treatment supporting virological control in people living with HIV (PLHIV). TB services and research in South Africa have been heavily impacted by recent US funding cuts. South Africa’s high TB burden and established service and research infrastructure have attracted significant US investments. The efforts to curb TB and TB-HIV co-infection were the result of carefully constructed partnerships between the National TB Programme (NTP), provincial health departments and development partners such as The President's Emergency Plan for AIDS Relief (PEPFAR), the US Centers for Disease Control and Prevention (CDC), The Global Fund, the Bill & Melinda Gates Foundation and, not least, the US Agency for International Development (USAID). In recent weeks, the US government first paused, then terminated foreign assistance through USAID. Though lacking the same finality, CDC support has also been largely affected. Uncertainty about future support poses a serious threat to sustaining these efforts beyond a mere reduction in funding. South Africa has one of the highest global burdens of TB, TB-HIV, and multidrug-resistant or rifampicin-resistant TB (MDR/RR-TB), with an estimated TB incidence of 427 per 100,000 population (range 265–626).^1^ In 2023, 270,000 new TB diagnoses were reported, more than half among PLHIV, and MDR/RR-TB incidence was estimated at 13,000.^1^

The South African TB Think Tank, whose membership accounts for a large number of TB service providers, non-governmental organisations (NGOs) and research teams nationwide, carried out a survey to document the impact of the withdrawal of funding on TB services and research. The survey findings highlight the significant instability TB services and research have faced since the 90-day stop-work orders were issued in late January 2025,^2^ affecting all programmes funded by CDC, USAID and PEPFAR. This was followed by the termination of all USAID-funded programmes in South Africa in late February.^3^ An additional US executive order on 7 February 2025 to halt financial aid to South Africa^4,5^ has led to further uncertainties, even where TB-related funding (e.g. through CDC) has been temporarily reinstated.^6^ US withdrawal from the WHO and other United Nations agencies has exacerbated this uncertainty and will have significant and sustained health impacts.^7,8^

For 2024/2025, South Africa’s NTP budget is US$244 million, US$164 million (67%) of which is domestically funded. The Global Fund contributes US$46 million (19%), and PEPFAR and USAID provide US$18 and 16 million, respectively, making up 14% of the total TB spend. Our early estimates show a net loss to prevention (US$12 million), screening (US$5 million), testing ($10 million) and treatment ($7 million). Without intervention, we estimate a risk of 580,000 fewer people being tested for TB and 35,000 fewer people receiving TB treatment in 2025. In the South African TB Think Tank survey, members were requested to provide data on the impact of funding cuts and work stoppages. Among 31 organisations contacted, 24 responded with 13 directly impacted thus far. Ten organisations identified a total of 22 grants affecting TB service provision, including 11 USAID grants totalling more than US$100 million. Affected organisations reported a median of 44% (IQR 25–90) of activities suspended, including linkage to TB care, health systems strengthening, TB prevention, screening, and testing, TB treatment and follow-up, community-based services, technical support to the Department of Health and TB-HIV integration. The Figure demonstrates which services are no longer offered through one NGO implementing USAID’s ACCELERATE 1 programme. These statistics do not capture the full impact on the NTP and services for people with TB. For instance, South Africa’s EDR (electronic data reporting) Web system for MDR/RR-TB, was almost entirely funded by USAID and now lacks support, putting the accuracy of TB reporting and follow-up at risk. The EDR Web system is critical for managing MDR/RR-TB, ensuring clients receive necessary care and tracking TB transmission. Without continued funding, the government must seek alternative support, which is concerning given the significant public health risks posed by MDR/RR-TB. Additionally, more than a third of the country's digital chest X-ray machines, crucial for early screening of high-risk populations, have been halted, increasing the risk of delayed detection and transmission. Aid agency furloughs and lay-offs have led to knock-on effects to publicly funded provincial TB programmes, resulting in delayed data capturing, thus affecting timely TB diagnosis and treatment initiation.

**Figure.**
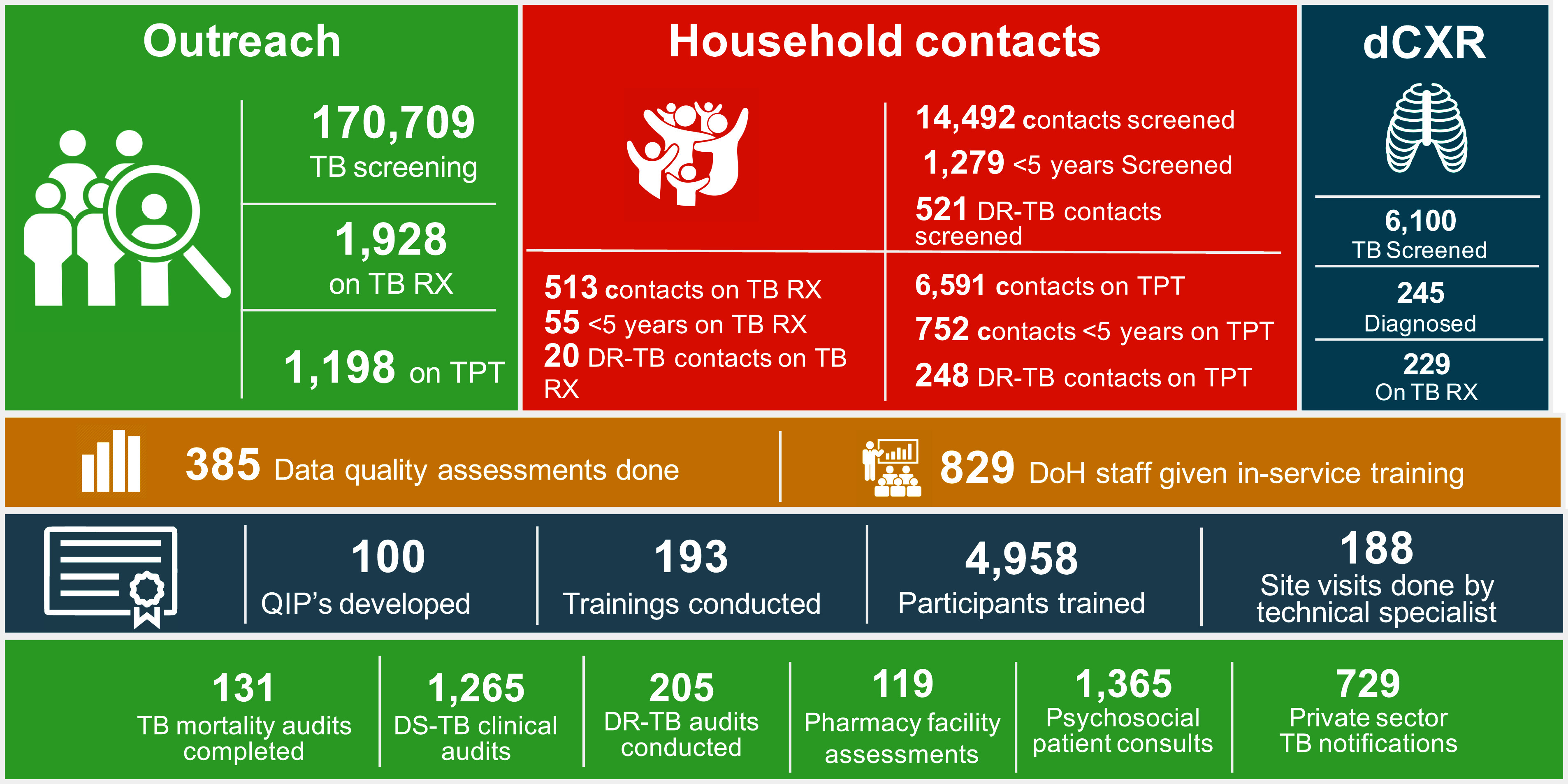
Services provided by The Aurum Institute implementing USAID’s ACCELERATE 1 programme in 2024, which will no longer be available. TB Rx = initiated on TB treatment; TPT = initiated on TB preventive treatment; DR-TB = drug-resistant TB; dCXR = digital chest X-ray; DoH = Department of Health; QIP = quality improvement programme; DS-TB = drug-susceptible TB.

In terms of research, eight organisations report a total of 14 grants affected to date, ranging from US$50,000–US$5 million per organisation for 2025 alone. Grants from the US National Institutes of Health (NIH) held by many experts across the country have not been withdrawn; however, some researchers have reported delays in funding renewals and others have been asked to justify the impact of their research beyond South Africa. This raises concerns that NIH funding may be revoked for the first time in history due to political interference. The implications for ongoing drug trials to improve drug-susceptible and MDR/RR-TB treatment are likely to be highly detrimental. Although the magnitude of these cuts is yet to be fully understood, the impact is already significant. This fallout extends to the rest of the world, including the United States, and will set back the global TB response. Delayed diagnosis and treatment initiation will fuel ongoing transmission, which together with increased drug resistance from interrupted clinical trials and treatment will have consequences that extend far beyond the borders of any one country.

In conclusion, the suspension of aid through the CDC and termination of many programmes through USAID and PEPFAR have had significant consequences for South African TB services and research. Funding disruptions have already hindered essential TB prevention, treatment and research efforts, threatening progress in reducing TB incidence and mortality. Uncertainty surrounding the future of US aid to TB services and research threatens to undermine the significant progress made in South Africa over the past decade. It also puts global advances, including those in the United States, at risk. A key example is the EDR Web system, which is vital to the country’s efforts in controlling drug-resistant TB. Its continued funding is crucial, and the urgent need for a global commitment to support such systems cannot be overstated. These disruptions highlight the responsibility of the global North and South to ensure sufficient resources are available for the global TB response.
